# MetaCancerDB: a database of site-specific RNA–miRNA correlations in cancer metastasis

**DOI:** 10.1093/database/baag026

**Published:** 2026-05-19

**Authors:** Myeonghun Cho, Byungkyu Park, Kyungsook Han

**Affiliations:** Department of Computer Engineering, Inha University, Inha-ro, Incheon 22212, Republic of Korea; Research Institute, JS Link Inc., Seoul 07793, Republic of Korea; Department of Quantum Information, Yonsei University, Seoul 03722, Republic of Korea; Department of Computer Engineering, Inha University, Inha-ro, Incheon 22212, Republic of Korea

## Abstract

Cancer metastasis involves complex molecular mechanisms that cannot be fully explained by individual gene expression profiles. Previous studies have shown that correlations between RNA and miRNA expression can capture metastatic behaviour more effectively than expression of individual genes. However, no publicly available databases provide systematic analysis of RNA–miRNA correlations specific to cancer metastasis. We developed an efficient computational method to identify differential correlations between miRNAs and RNAs that are specific to individual tumour samples. Using data from The Cancer Genome Atlas (TCGA), we computed differential correlations for tumour samples across 9 cancer types and 21 metastatic sites, encompassing ~200 million RNA–miRNA pairs. Statistical analysis identified RNA–miRNA pairs with site-specific correlations using Mann–Whitney *U*-tests. MetaCancerDB contains RNA–miRNA correlation networks for 9 primary cancer types and 21 metastatic sites. Site-specific correlations showed distinct patterns, with lung metastasis displaying the most conserved correlations across cancer types. Survival analysis revealed that specific RNA–miRNA pairs are prognostic for patient outcomes in a metastatic site-dependent manner. MetaCancerDB provides a comprehensive resource for exploring RNA–miRNA correlations in cancer metastasis. The database enables researchers to identify molecular signatures specific to metastatic sites and can serve as a foundation for developing predictive biomarkers. MetaCancerDB is freely available for academic purposes.

**Database URL:**  https://metacancerdb.hpid.org

## Introduction

Despite significant advances in cancer research and treatment, cancer remains one of the most deadly diseases. By 2050, there are projected to be >35 million new cases of cancer worldwide, an increase of 77% from the 20 million cases reported in 2022 [1]. Approximately 90% of cancer-related deaths are attributed to metastasis [2], which is the spread of tumour cells from the primary site to other parts of the body typically through the lymphatic system or blood. Thus, predicting metastasis risk is important when determining treatment options for cancer patients.

Motivated by a large amount of transcriptome data in cancer cells generated by RNA sequencing or microarray, several computational methods have been developed to predict cancer metastasis from gene expression data. For instance, Albaradei *et al*. [3] developed a deep learning model called MetastaSite, which predicts whether a tumour sample is primary or metastasized to other distant organs using gene expression data. A support vector machine (SVM) model developed by Zhang *et al*. [4] predicts lymph node metastasis based on differentially expressed mRNAs and non-coding RNAs in cancer. A graph convolution neural network model called GCNN-Kirchhoff [5] predicts multiple metastatic sites of breast cancer by integrating multiomics data into a knowledge graph. Zhou *et al*. [6] proposed an algorithm called PLUS (Positive and unlabelled Learning from Unbalanced cases and Sparse structures) to predict distant metastasis potential and found 191 metastasis-predictive genes using PLUS.

All of these methods used gene expression data and/or multiomics data to predict lymph node metastasis or distant metastasis, and did not consider correlation of genes. Cancer is a complex disease and so abnormal expression of individual genes cannot fully explain the development of cancer and metastasis. Dysregulated gene interactions are known to be related to cancer. As an example, a gene regulation mechanism known as competing endogenous (ceRNA) hypothesis [7] suggests that RNAs with similar miRNA response elements compete to bind to the same miRNA, thereby regulating each other indirectly. There is an increasing evidence which supports the hypothesis. For instance, miR-138 binding to *AKT1* regulates the expression of *AKT1* in tongue squamous cell carcinoma [8], and miR-519d inhibits lymph node metastasis by regulating *MMP3* in oral squamous cell carcinoma and breast cancer [9, 10].

Inspired by the ceRNA hypothesis, we previously computed differential correlations between RNAs and miRNAs specific to individual tumour samples, and used the differential correlations in predicting metastasis or prognosis. Distant metastasis is much harder to predict than lymph node metastasis partly due to the small number of tumour samples for which information about distant metastasis is known. Predicting distant metastatic sites is even more challenging than predicting whether or not distant metastasis will occur. This is because the problem of predicting distant metastatic sites is a multi-class and multi-label classification problem: there are more than two classes of distant metastatic site (e.g. bone, brain, liver, and lung), and a single sample can have multiple labels for multiple metastatic sites. Our previous studies showed that gene correlations are more powerful and reliable features than expressions of individual genes when predicting metastasis or metastatic sites [11–13].

There are few databases that consolidate transcriptome data at primary sites and metastatic sites in a consistent format, so collecting raw data from disparate sources and processing the data is a significant burden for researchers. The Human Cancer Metastasis Database (HCMDB) [14] is one of the few databases that provides transcriptome data for both primary and metastatic cancers. It enables exploration of metastasis-associated genes and co-expression networks of mRNAs and lncRNAs, but its functionality is intended for the analysis at the cohort level rather than at the individual patient level. cBioPortal [15] enables access to genomic alterations, gene expression, and clinical data at the individual patient level, but as a general purpose database it lacks functionality for metastatic diseases and provides a limited number of metastasis-related samples. For example, only about 2% of the breast cancer patients (22 individuals) of The Cancer Genome Atlas (TCGA) [16] were classified as having distant metastasis in cBioPortal. Moreover, metastatic events and their anatomical locations are missing in cBioPortal, so conducting metastasis research at the individual patient level using cBioPortal is not easy.

The gene correlations computed by our previous studies are for developing predictive models of metastasis of several types, so are not convenient for external researchers to use. We recently constructed a database called MetaCancerDB to help researchers and clinicians examine metastasis at the individual patient level. For each cancer patient, it provides transcriptome data at both primary and metastatic sites as well as gene correlations specific to the patient. MetaCancerDB can also be used as a useful resource when developing predictive models of metastasis. The rest of this paper presents the way we constructed MetaCancerDB and an example of using it.

## Methods

### Data collection and preprocessing

For all samples of 33 cancer types in TCGA, we collected clinical data, including diagnoses and follow-up information, from the National Cancer Institute (NCI) Genomic Data Commons (GDC) Data Portal (Data Release 43.0, released on 7 May 2025) [17]. Samples were first classified into normal and tumour based on the sample type in the diagnoses data, and tumour samples were further classified using the Tumour, Node, and Metastasis (TNM) staging system. The diagnosis data include information on metastatic sites for some samples for which metastasis was detected at the initial diagnosis. But, for many samples in which metastasis events occurred later in the disease course, information such as metastasis event and metastatic sites is missing in the diagnosis data. To supplement missing or incomplete metastasis records, we used the follow-up data as well. In the follow-up data, we extracted the progression_or_recurrence_anatomic_site column for the information on metastatic sites. We used the definitions of the GDC to ensure consistency in the naming of cancer metastasis sites.

We selected cancer types which satisfy the following criteria: (1) metastatic site information was available in the follow-up data, (2) at least 10 normal samples were present, and (3) at least one metastatic site included five or more samples. Among the 33 cancer types in TCGA, 9 cancer types met the selection criteria.

We obtained expression data for RNAs and miRNAs from the TCGA database. For mRNA expression, we utilized Transcripts Per Million (TPM) normalized values, which account for both sequencing depth and gene length. For miRNA expression, we performed Counts Per Million (CPM) normalization using raw sequencing counts. CPM was preferred for miRNAs because their relatively uniform and short sequence length (typically 18–25 nucleotides) makes length-based normalization, such as TPM, less critical while maintaining a comparable scale with TPM values. We used miRNA-seq data instead of RNA-seq data because most miRNA expression levels in RNA-seq data are close to zero due to the short sequence length of miRNAs. RNAs and miRNAs with zero variance in expression among normal samples were excluded in our database.

Additionally, we required that both RNA-seq and miRNA-seq data were available for the same biological samples to enable correlation analysis. Each sample of TCGA is labelled with a structured barcode and we used the portion up to the vial level (e.g. TCGA-AB-1234-01A) to match RNA-seq and miRNA-seq data. This ensured that both data types originated from the same biological specimen. When multiple portions were available for a given sample, we prioritized those not subjected to formalin-fixed, paraffin-embedded (FFPE) processing. If all available portions were FFPE or lacked FFPE information, we selected the one with the alphabetically earliest portion identifier to maintain consistency across samples. Table 1 shows the number of normal samples, total tumour samples, tumour samples with metastasis, RNAs, miRNAs, and metastatic sites in tumour samples.

**Table 1:** Summary of data in MetaCancerDB.^a^

	Samples (*n*)				Genes (*n*)	
Primary cancer	Normal	Total tumour	Metastatic tumour	Metastatic sites (*n*)	RNA	miRNA
BLCA	19	406	208	6	47 416	706
BRCA	104	1087	585	6	55 019	1306
ESCA	13	183	128	5	55 244	541
HNSC	44	515	305	7	50 718	1206
LIHC	50	367	169	2	47 951	1215
PRAD	52	497	85	1	52 328	1006
STAD	36	409	296	4	54 670	940
THCA	59	504	243	2	53 100	1441
UCEC	33	539	100	6	51 858	1091

a Numbers show sample counts for normal samples, total tumour samples, tumour samples with metastasis information, distinct metastatic sites, RNA genes, and miRNAs for each cancer type. Metastatic site count refers to distinct anatomical locations with metastasis information in follow-up data.

### Computing gene correlations efficiently

In our previous studies, we computed gene correlations in the following way. For every pair of RNAs and miRNAs in *n* normal samples, we computed the Pearson correlation coefficient (PCC) between their expression levels in Equation (1). In the equation, $\bar{X}$ and $\bar{Y}$ represent the mean of *X* and mean of *Y*, respectively. After adding a single tumour sample to the *n* normal samples, we computed $PCC_{n+1}$ in $n+1$ samples. We then computed the change in PCC (i.e. $\Delta$PCC) for every RNA–miRNA pair by subtracting $PCC_n$ from $PCC_{n+1}$ (Equation 2). $\Delta$PCCs reflect the differences in gene correlations between the normal samples and the tumour sample. Since the differences are due to the added tumour sample, different tumour samples show different $\Delta$PCCs, which make it possible to derive gene correlations specific to each tumour sample.


(1) \begin{eqnarray*}
PCC_n(X,Y) = \frac{\sum \limits _{i=1}^{n}(X_i - \bar{X})(Y_i - \bar{Y})}{\sqrt{\sum \limits _{i=1}^{n}\left(X_i-\bar{X_n}\right)^2}\sqrt{\sum \limits _{i=1}^{n}\left(Y_i-\bar{Y_n}\right)^2}}
\end{eqnarray*}

(2) \begin{eqnarray*}
\Delta PCC(X,Y) = PCC_{n+1}\left(X,Y\right) - PCC_{n}\left(X,Y\right)
\end{eqnarray*}

However, this method of computing $\Delta$PCCs is not computationally efficient because it requires the full set of RNA and miRNA expression values even though a same set of normal samples is used for all tumour samples. For cancer types with a large number of normal samples, such as BRCA, this leads to significant memory usage and redundant computation.

Thus, we improved the computation process as follows. Instead of computing PCC for each RNA–miRNA pair, we reformulated it in a matrix form, allowing us to compute correlations for all pairs simultaneously. Furthermore, rather than retaining the full matrix for normal samples in memory, we precalculated the matrix and stored essential statistics, such as the mean and sum of squared deviations. The improved calculation of $\Delta$PCC is described in Equation (3), where $\bar{X_n}$, $\bar{Y_n}$, $SS_{X_n}$, $SS_{Y_n}$, and $CN_n$ denote the mean of RNA X, mean of miRNA Y, sum of squared deviations of X, sum of squared deviations of Y, and covariance numerator in *n* normal samples, respectively. These values are precalculated once with normal samples. The new method significantly reduced memory and time requirements, allowing computation of $\Delta$PCCs of all RNA–miRNA pairs in all tumour samples of various types of cancer.


(3) \begin{eqnarray*}
PCC_{n+1}(X,Y) = \frac{\sum \limits _{i=1}^{n+1} \left( X_i - \bar{X_{n+1}} \right) \left(Y_i - \bar{Y_{n+1}} \right)}{\sqrt{\sum \limits _{i=1}^{n+1} \left(X-\bar{X_{n+1}} \right)^2}\sqrt{\sum \limits _{i=1}^{n+1} \left(X-\bar{X_{n+1}} \right)^2} } \\=\frac{CN_n + (X_{n+1} - \bar{X_n})(Y_{n+1} - \bar{Y_n})}{\sqrt{SS_{X_n} + \frac{n}{n+1}\left(X_{n+1} - \bar{X_n} \right)^2 }\sqrt{SS_{Y_n} + \frac{n}{n+1}\left(Y_{n+1} - \bar{Y_n} \right)^2} }
\end{eqnarray*}

where


\begin{eqnarray*}
SS_{X_n} &=& \sum \limits _{i=1}^{n}\left(X_i-\bar{X_n} \right)^2 \\SS_{Y_n} &=& \sum \limits _{i=1}^{n}\left(Y_i-\bar{Y_n} \right)^2 \\CN_n &=& \sum \limits _{i=1}^{n} \left(X_i - \bar{X_n} \right)\left(Y_i - \bar{Y_n}\right)
\end{eqnarray*}

The time complexity of computing $\Delta$PCCs was reduced from *O*(t*n*k) to *O*(t*k), and the space complexity was reduced from *O*(n*k) to *O*(*k*) in each cancer type, where *n* is the number of normal samples, *t* is the number of tumour samples, *k* is the number of RNA–miRNA pairs. This improvement enabled processing of ~200 million RNA–miRNA pairs in thousands of samples much more efficiently than before.

### Identifying RNA–miRNA pairs with correlations specific to a metastatic site

To identify RNA–miRNA correlations specific to every metastatic site, we performed statistical testing on $\Delta$PCCs. For each cancer type, tumour samples with metastasis were divided into two groups: (1) samples with metastasis to a specific site and (2) samples without metastasis to that site. Since $\Delta$PCCs did not show normal distribution, we determined whether there are significant differences in $\Delta$PCC between the two groups using the Mann–Whitney *U*-test. The test was conducted for metastatic sites with at least five tumour samples metastasized to the sites. RNA–miRNA pairs with a *P*-value less than .001 in the Mann–Whitney *U*-test were considered significant. We prioritized a stringent raw *P*-value threshold over universal multiple testing corrections (e.g. FDR) to ensure the inclusion of rare metastatic sites. Given the large-scale testing of >33 million pairs, conservative corrections would impose a mathematical significance threshold (~ $10^{-9}$), which small cohorts (e.g. *n*=5) cannot realistically achieve, even with perfect rank separation. By maintaining this threshold, we aimed to prevent clinical bias towards prevalent cancer types and minimize Type II errors (false negatives), thereby providing a comprehensive candidate pool for exploratory discovery and subsequent experimental validation.

## Results and discussion

### RNA–miRNA pairs with correlations specific to a metastatic site

In total, we calculated ~500 million $\Delta$PCCs of RNA–miRNA across 9 cancer types. Table 2 shows significant RNA–miRNA pairs in 9 types of cancer and 19 metastatic sites. Details of the pairs, including their $\Delta$PCCs, are available on the MetaCancerDB download page.

**Table 2:** Number of tumour samples and RNA–miRNA pairs with correlations specific to a metastatic site.^a^

Metastatic site	BLCA	BRCA	ESCA	HNSC	
Abdomen	*	0	0	0	
Bone	36 (106 018/97 090)	40 (51 984/51 126)	5 (1716/1693)	*	
Brain	*	5 (2122/2089)	8 (64 970/63 756)	*	
Floor of mouth	0	0	0	6 (30 224/29 853)	
Head, face, or neck lymph nodes	0	0	*	26 (73 680/72 686)	
Intra-abdominal lymph nodes	0	0	0	0	
Intra-thoracic lymph nodes	0	5 (3964/3908)	*	*	
Lung	40 (27 510/26 167)	18 (157 622/155 137)	11 (182 273/178 438)	37 (524 982/522 374)	
Liver	21 (13 813/13 153)	17 (32 134/31 393)	20 (116 234/112 015)	*	
Lymph node	35 (26 053/24 852)	*	5 (24 675/24 391)	0	
Mouth	0	0	0	19 (106 172/104 542)	
Oropharynx	0	0	0	10 (17 134/16 744)	
Pelvis	17 (42 388/40 246)	0	0	*	
Peritoneum	*	0	*	0	
Renal pelvis	7 (27 677/25 577)	0	0	0	
Soft tissue of head and neck$^{1}$	0	0	0	5 (8440/8372)	
Thorax	0	7 (215 154/212 496)	0	*	
Tongue	0	0	0	10 (15 505/15 302)	
Vagina	*	0	0	0	
**Metastatic site**	**LIHC**	**PRAD**	**STAD**	**THCA**	**UCEC**
Abdomen	*	0	0	0	9 (52 051/52 014)
Bone	9 (40 530/40 227)	7 (3 017/3 017)	*	*	5 (770/769)
Brain	*	0	*	0	*
Floor of mouth	0	0	0	0	0
Head, face, or neck lymph nodes	0	0	*	0	0
Intra-abdominal lymph nodes	*	0	0	0	6 (5275/5265)
Intra-thoracic lymph nodes	0	0	0	0	0
Lung	18 (42 057/41 805)	*	11 (58 206/58 012)	16 (516 976/507 763)	11 (34 210/34 146)
Liver	117 (–)	0	30 (378 768/378 507)	*	8 (8871/8842)
Lymph node	*	*	6 (3611/3583)	22 (813 487/804 274)	*
Mouth	0	0	0	0	0
Oropharynx	0	0	0	0	0
Pelvis	0	0	0	0	*
Peritoneum	*	0	5 (3867/3810)	0	*
Renal pelvis	0	0	0	0	0
Soft tissue of head and neck$^{1}$	0	0	0	0	0
Thorax	0	0	0	0	*
Tongue	0	0	0	0	0
Vagina	0	0	0	0	16 (58 312/58 225)

a Each cell shows the number of tumour samples, followed by two values in parentheses: the total number of significant RNA–miRNA pairs identified at the metastatic site and the number of RNA–miRNA pairs unique to the metastatic site in the same primary cancer type. RNA–miRNA pairs were computed only for cancer–site combinations with at least five tumour samples. An asterisk (*) indicates non-zero but fewer than five samples. Soft tissue of head and neck^1^: connective, subcutaneous, and other soft tissues of head, face, and neck.

Naturally, there were very few RNA–miRNA pairs common to different metastatic sites. In the survival analysis with respect to the RNA–miRNA pairs, we observed that some site-specific RNA–miRNA pairs are related to the patient’s prognosis in the presence of metastasis to the site, but not at other sites.

Correlation changes such as $\Delta$PCCs in RNA–miRNA pairs in individual cancer patients are powerful characteristics to predict distant metastatic sites. In our previous study [12], we compared our model that uses differential correlations of RNA–miRNA pairs with two other methods: MetastaSite [3] and GCNN-Kirchhoff [5]. MetastaSite is a multi-class deep neural network (DNN) to classify primary cancer samples and those metastasized samples, whereas GCNN-Kirchhoff is a graph convolutional neural network (GCNN) combined with Kirchhoff’s law to predict metastatic sites. As shown in Table 3, our model, which uses $\Delta$PCCs of RNA–miRNA pairs, achieved much better performance than the others.

**Table 3:** Performance of our model [12], MetastaSite [3], and GCNN-Kirchhoff [5] in predicting metastatic sites.^a^

	Bone	Liver	Lung
Our model	0.94	0.98	0.93
MetastaSite	0.72	0.61	0.78
GCNN-Kirchhoff	0.77	0.56	0.68

a Comparison was made in terms of the Area Under the Curve (AUC) values. Our model used PCCs of RNA–miRNA pairs as features.

### Overall statistics of identified RNA–miRNA pairs

Across all 9 cancer types and 19 metastatic sites, we identified a total of 3 892 451 significant RNA–miRNA pairs (average of 204 866 pairs per metastatic site). These pairs showed a *P*-value < .001 in the Mann–Whitney *U*-test. The distribution varied considerably across cancer types, with THCA showing the highest number of site-specific pairs (average of 665 232 pairs per metastatic site) and PRAD showing the lowest (average of 3017 pairs per site).

Lung metastasis was associated with the largest number of RNA–miRNA pairs across multiple cancer types, with significant correlations identified in eight out of nine cancer types. In contrast, brain metastasis showed highly specific correlations limited to BRCA and ESCA.

**Figure 1:**
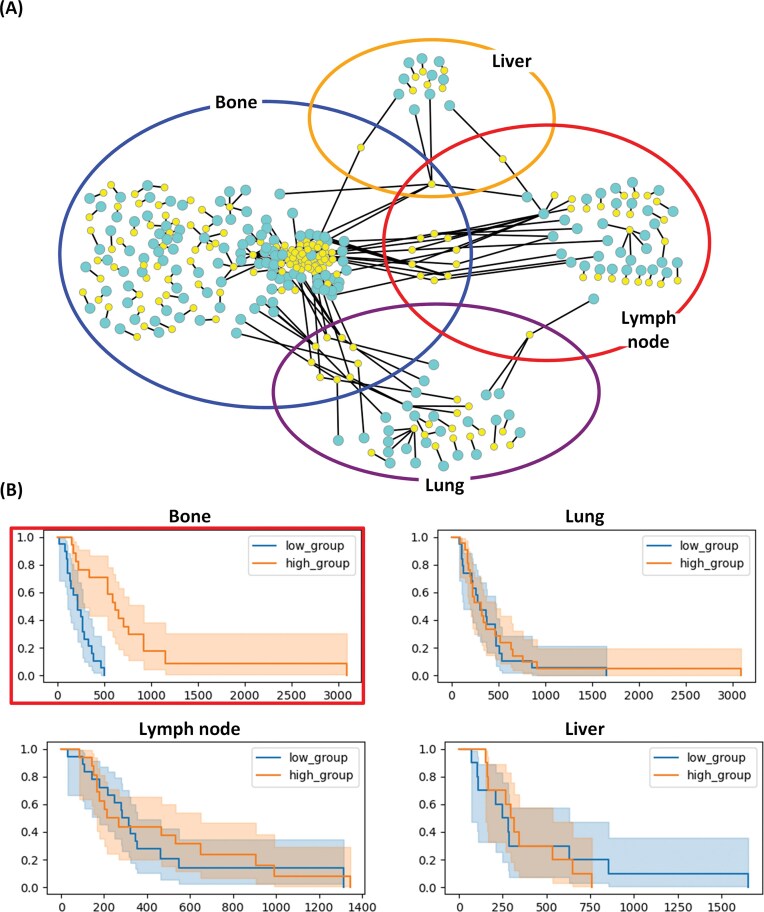
RNA–miRNA correlation networks specific to four metastatic sites of BLCA. (A) Subnetworks show top-ranked RNA–miRNA pairs (*P*<.001) specific to bone (blue), liver (yellow), lung (purple), and lymph node (red) metastases. Yellow nodes in the networks represent miRNAs and cyan nodes represent RNAs. (B) Kaplan–Meier plots for *RALGAPB*-hsa-miR-224-5p correlation in different metastatic sites. Patients were stratified by median $\Delta$PCC value. The correlation is prognostic for bone metastasis (*P*<.001 in the log-rank test) but not for other metastatic sites.

Figure 1A shows the network of RNA–miRNA pairs in BLCA, where each subnetwork consists of RNA–miRNA pairs specific to one of four metastatic sites (bone, liver, lung, and lymph node). For each of the RNA–miRNA pairs in the four subnetworks, we performed the survival analysis with respect to $\Delta$PCC of the pair. Some RNA–miRNA pairs were prognostic when metastasis occurred at the metastatic site for which the RNA–miRNA pairs were derived.

As an example, the four Kaplan–Meier (KM) plots in Fig. 1B show the survival rates with respect to $\Delta$PCC (*RALGAPB*, hsa-miR-224-5p) in the bone metastasis network of Fig. 1A. hsa-miR-224-5p in the pair is an miRNA with the highest degree in the network of RNA–miRNA pairs in BLCA. The pair of *RALGAPB* and hsa-miR-224-5p. $\Delta$PCC of *RALGAPB* and hsa-miR-224-5p was effective in predicting survival rates of patients with bone metastasis (top left KM plot enclosed in a red box). There is a significant difference (*P*-value < .001) between the two groups of patients with bone metastasis. Patients with a higher $\Delta$PCC (*RALGAPB*, hsa-miR-224-5p) than the median $\Delta$PCC showed much higher survival rates than patients with lower $\Delta$PCC (*P*-value < .001). But the pair was predictive only in patients with bone metastases, and not predictive in patients with other metastases.

Gene ontology analysis of the RNA components in site-specific pairs revealed enrichment in pathways related to cell migration, invasion, and epithelial-mesenchymal transition (EMT). For example, *RALGAPB* identified in bone metastasis of BLCA is involved in small GTPase signalling pathways known to regulate cell motility and invasion [18, 19]. hsa-miR-224-5p, which showed the highest connectivity in the BLCA network, has been previously implicated in cancer progression and is known to regulate multiple oncogenic pathways [20].

### RNA–miRNA pairs common to different cancer types

**Figure 2:**
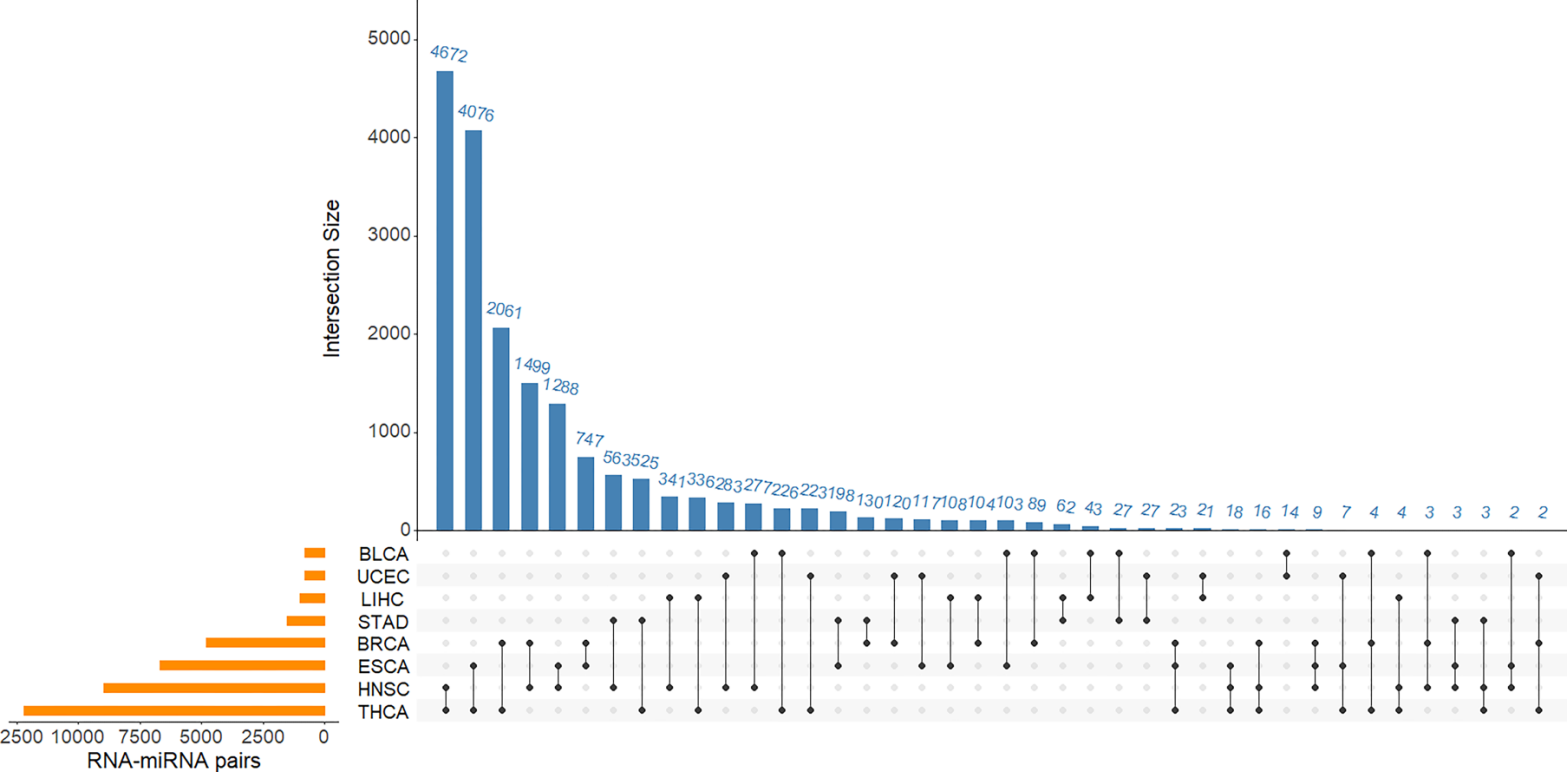
Lung metastasis associated RNA–miRNA pairs common to different primary cancer types. The orange bars in the left panel show the number of RNA–miRNA pairs from each primary cancer type. The blue bars in the top panel show the number of common pairs in each combination of primary cancer types, while the black dots bottom panel show the primary cancer types involved in the intersection.

In this study, we derived RNA–miRNA pairs with correlations specific to every metastatic site, and found that different metastatic sites share a very small number of such RNA–miRNA pairs. In contrast, many RNA–miRNA pairs with site-specific correlations were found to be common to several primary cancer types. Figure 2 displays the intersection of RNA–miRNA pairs between different primary cancer types using an UpSet plot. The RNA–miRNA pairs were derived as pairs specific to lung metastasis. The orange bars in the left panel show the number of RNA–miRNA pairs from each primary cancer type. The blue bars in the top panel show the number of common pairs in each combination of primary cancer types, while the black dots bottom panel show the primary cancer types involved in the intersection.

### Construction and using MetaCancerDB

We implemented MetaCancerDB with a React (https://react.dev) frontend using Vite (https://vite.dev) and a FastAPI backend (https://fastapi.tiangolo.com). We used PostgreSQL (https://www.postgresql.org) for managing and storing data of MetaCancerDB.

**Figure 3:**
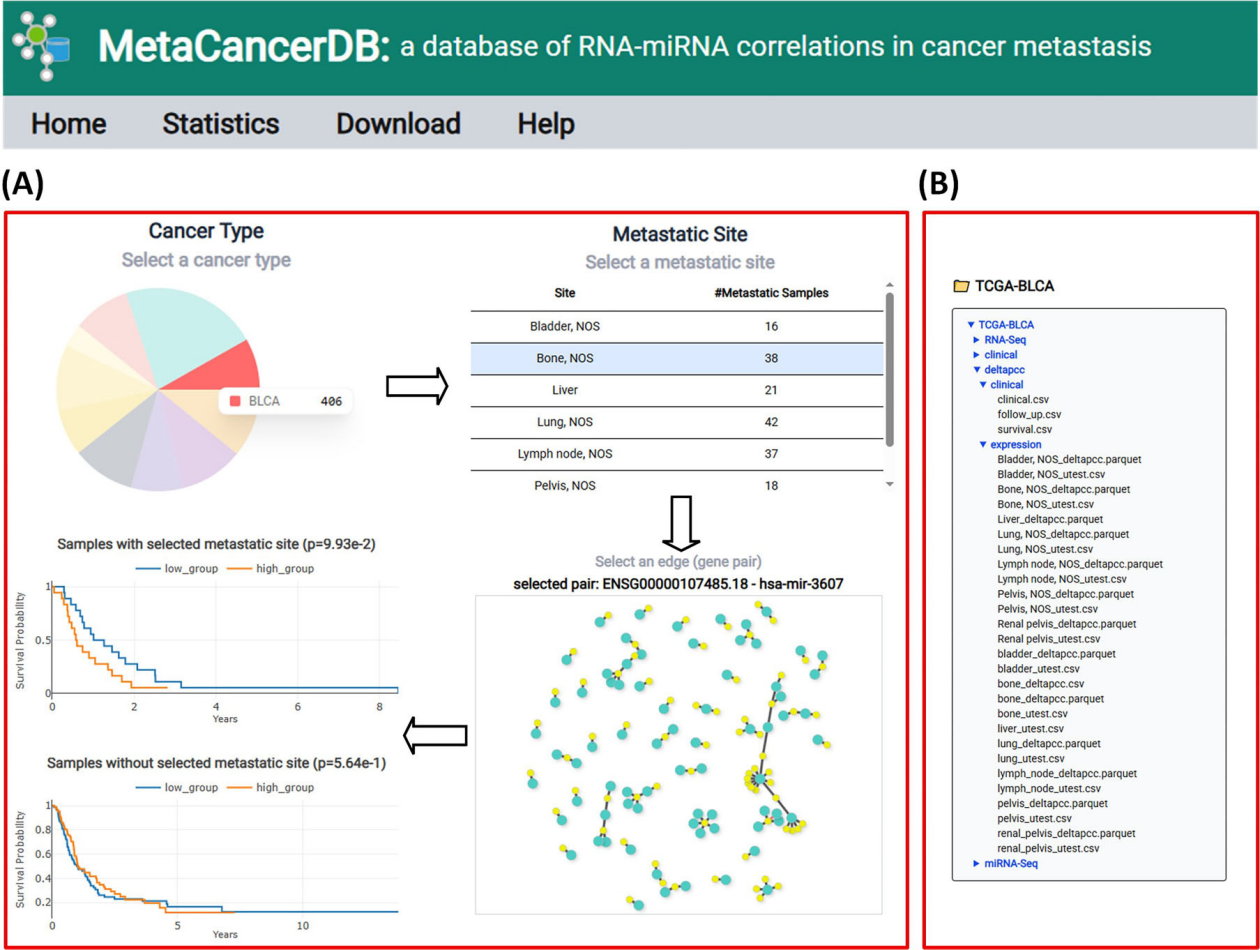
Example of using MetaCancerDB. (A) The Cancer Type panel visualized in a pie chart shows the primary cancer types in MetaCancerDB. The number of samples of each cancer type is displayed when users move a mouse over a cancer type. When users click a primary cancer type, a table of metastatic sites of the cancer type is displayed along with the number of metastatic samples at the sites. For a selected metastatic site (a row in the table), a network consisting of top 100 RNA–miRNA pairs ranked by their *P*-values in correlations is visualized. When users move a mouse over a node or edge, detailed information on the node or edge is displayed. Clicking an edge highlights the selected edge in red, and two Kaplan–Meier plots are generated for the selected pair, which compares survival rates of patient groups. The upper plot shows the survival rates of patients of the selected primary cancer type and with metastasis at the selected site, while the lower plot shows the survival rates of patients of the same primary cancer type but without metastasis at the selected site. (B) Example of the download page. The database provides all processed data, including RNA-seq, miRNA-seq, $\Delta$PCC of RNA–miRNA pairs, follow-up data, survival, and clinical data. Each type of data is downloadable by clicking its entry in the file list.

In the MetaCancerDB platform, users can interactively explore a network of RNA–miRNA pairs by first selecting a primary cancer type in a pie chart, and then a metastatic site of the selected cancer type in a table (Fig. 3A). After selecting the cancer type and metastatic site, a network consisting of top 100 RNA–miRNA pairs with low *P*-values in $\Delta$PCC is visualized. In the network, each node represents an RNA or miRNA molecule, and each edge represents an RNA–miRNA pair. When users move a mouse over a node or edge, detailed information on the node or edge is displayed.

When users click an edge, two KM plots are visualized in real time for the selected RNA–miRNA pair. The upper KM plot shows the survival analysis with respect to $\Delta$PCC of the pair in patients who had metastasis in the selected site. The lower KM plot displays the survival analysis in patients who did not have metastasis in the site. This dual-view approach enables users to assess the prognostic significance of the RNA–miRNA pair. In addition to the top-ranked RNA–miRNA pairs included in the network, all other RNA–miRNA pairs and their correlations are available in the MetaCancerDB download page.

The site-specific RNA–miRNA correlations identified in MetaCancerDB have potential clinical applications. The prognostic value demonstrated for certain pairs (e.g. *RALGAPB*-hsa-miR-224-5p in BLCA bone metastasis) suggest these correlations could be developed into biomarkers for patient stratification and treatment planning.

Furthermore, the identification of common RNA–miRNA pairs across different primary cancer types metastasizing to the same site suggests shared molecular mechanisms that could be targeted therapeutically. This finding supports the concept of metastasis site-specific rather than primary tumour-specific therapeutic approaches.

## Limitations and future work

Several limitations should be acknowledged. First, our analysis is limited to TCGA data, which do not cover all types or populations of cancer. To validate our findings, we searched for others using the following criteria: (1) paired RNA and miRNA expression data, (2) annotation of metastatic sites, and (3) normal samples available at each metastatic site. While there are many single-omic datasets, we could not find public repositories that satisfy all these requirements simultaneously. Due to the data scarcity in multi-omic metastatic profiles, we could not perform a large-scale independent validation. Second, the correlation analysis assumes linear relationships between RNA and miRNA expression, while biological interactions may be more complex. Third, metastatic site information relies on clinical annotations, which may have inconsistencies or missing data. Future work could include integration of additional datasets, incorporation of non-linear relationship models, and experimental validation of identified RNA–miRNA pairs.

## Conclusion

miRNAs interacting with RNAs often regulate gene expression, and correlations between RNAs and miRNAs can capture metastatic behaviour more effectively than expression profiles of individual genes. So far, there are no publicly available databases which provide correlations between RNAs and miRNAs in cancer metastasis. In this study, we developed an efficient method of computing differential correlations between miRNAs and RNAs, which are specific to individual tumour samples, and computed differential correlations for all tumour samples of various cancer types. From an extensive analysis of the differential correlations in various cancer types and metastatic sites, we identified RNA–miRNA pairs with correlations specific to each metastatic site. This paper presented a database called MetaCancerDB, which was constructed for 9 primary cancer types and 21 metastatic sites. For a primary cancer type and metastatic site specified by a user, MetaCancerDB provides a network of RNA–miRNA pairs whose correlations are specific to the selected primary cancer type and metastatic site. Some RNA–miRNA pairs in the network were found to be predictive of survival rates in patients with metastasis in the selected site. MetaCancerDB can serve as a useful resource of differential correlations between RNAs and miRNAs in patients with metastasis. The correlations can be used as powerful features when predicting metastasis or metastatic sites. MetaCancerDB is freely available at https://metacancerdb.hpid.org
